# Systemic Multimorbidity Clusters in People with Periodontitis

**DOI:** 10.1177/00220345221098910

**Published:** 2022-06-09

**Authors:** H. Larvin, J. Kang, V.R. Aggarwal, S. Pavitt, J. Wu

**Affiliations:** 1 School of Dentistry, University of Leeds, Leeds, UK; 2 Oral Biology, School of Dentistry, University of Leeds, Leeds, UK; 3 Leeds Institute for Data Analytics, University of Leeds, Leeds, UK

**Keywords:** oral health, periodontal diseases, big data, epidemiological factors, hypergraph, network analysis

## Abstract

This study aimed to identify systemic multimorbidity clusters in people with periodontitis via a novel artificial intelligence–based network analysis and to explore the effect of associated factors. This study utilized cross-sectional data of 3,736 participants across 3 cycles of the National Health and Nutrition Examination Survey (2009 to 2014). Periodontal examination was carried out by trained dentists for participants aged ≥30 y. The extent of periodontitis was represented by the proportion of sites with clinical attachment loss (CAL)≥ 3 mm, split into 4 equal quartiles. A range of systemic diseases reported during the survey were also extracted. Hypergraph network analysis with eigenvector centralities was applied to identify systemic multimorbidity clusters and single-disease influence in the overall population and when stratified by CAL quartile. Individual factors that could affect the systemic multimorbidity clusters were also explored by CAL quartile. In the study population, the top 3 prevalent diseases were hypertension (63.9%), arthritis (47.6%), and obesity (45.9%). A total of 106 unique systemic multimorbidity clusters were identified across the study population. Hypertension was the most centralized disease in the overall population (centrality [C]: 0.50), followed closely by arthritis (C: 0.45) and obesity (C: 0.42). Diabetes had higher centrality in the highest CAL quartile (C: 0.31) than the lowest (C: 0.26). “Hypertension, obesity” was the largest weighted multimorbidity cluster across CAL quartiles. This study has revealed a range of common systemic multimorbidity clusters in people with periodontitis. People with periodontitis are more likely to present with hypertension and obesity together, and diabetes is more influential to multimorbidity clusters in people with severe periodontitis. Factors such as ethnicity, deprivation, and smoking status may also influence the pattern of multimorbidity clusters.

## Introduction

Multimorbidity is the notion of having ≥2 coexisting chronic conditions and is often associated with reduced quality of life and higher risk of mortality (Yarnall et al. 2017; Jani et al. 2019). Importantly, multimorbidity significantly affects health care services utilization, including issues with polypharmacy and uncoordinated care (Yarnall et al. 2017). While it is widely accepted that the prevalence of multimorbidity increases with age, findings suggest that other factors, such as deprivation and some chronic conditions, may influence multimorbidity development (Barnett et al. 2012). Understanding the clustering of multimorbidity in people with chronic conditions is important and can be integrated into patient care to improve patient outcomes. Studies of multimorbidity clusters have centered on major chronic conditions, such as cardiovascular disease or cancer (Zemedikun et al. 2018; Jani et al. 2019); however, the current evidence of multimorbidity clusters in people with periodontitis is scarce (Watt and Serban 2020).

Studies have shown an association between periodontitis and nonoral diseases, such as diabetes mellitus (diabetes; Preshaw et al. 2012), obesity (Suvan et al. 2011; Kang et al. 2019), and cardiovascular disease (CVD; Larvin et al. 2021b). These studies are typically limited to traditional epidemiological design with a single pre-defined outcome measured at a time and limits understanding of the associations within multimorbidity clusters. Data-driven studies use advanced techniques such as artificial intelligence and machine learning to reveal information behind the data, without the requisite of predefined outcomes. Through process mining a longitudinal data set, we recently showed that self-reported periodontitis might be a precursor to multimorbidity development in otherwise systemically healthy individuals (Larvin et al. 2021a). However, robust evidence based on clinical periodontal examination data of the typical systemic multimorbidity clusters in people with periodontitis is still lacking.

Hypergraph analysis is an artificial intelligence method based on the network analysis approach, which reveals possible clustering of characteristics in a population. This is a novel method to multimorbidity research and was outlined in a recent feasibility study (Rafferty et al. 2021). Eigenvector centrality is a measure that represents how influential diseases are to multimorbidity clusters—that is, how well “connected” they are to other diseases. Traditional regression analysis is limited to single predefined outcomes; multimorbidity research in this regard is therefore limited to pairwise methods that may reveal multimorbidity clusters that are not actually representative of the true population. The hypergraph technique allows for multiple conditions to be analyzed simultaneously, with tangible multimorbidity clusters identified from the data. The hypergraphs can visualize systemic multimorbidity clusters of any number of conditions and are distinct from classical network analysis graphs, which are limited to pairwise methods. This technique also enables quantification of centrality as a means of understanding which diagnoses are most central to the multimorbidity clusters. As precision or personalized medicine moves to the forefront of health research, it is important to profile distinct populations with multimorbidity, improving the understanding of how diagnoses may interact and inform subsequent clinical decisions.

In this study, we aim to identify systemic multimorbidity clusters in people with periodontitis by applying hypergraph analysis to data from the National Health and Nutrition Examination Survey (NHANES; Centers for Disease Control and Prevention 2022). Furthermore, we explore factors that may affect centrality of systemic multimorbidity clusters.

## Methods

### Study Design

This cross-sectional study is based on participants from the NHANES between 2009 and 2014.

### Database

Data from the NHANES were utilized in this analysis. The NHANES program is conducted over 2 y by the Centers for Disease Control and Prevention. The data set contains self-reported survey responses to questions related to demographics (age, sex, and ethnicity), socioeconomics (household income quintile), and health (smoking status, health conditions). Oral health information from clinical dental examinations and physiologic measurements such as body mass index and blood pressure from medical examinations by trained health professionals are also captured. Each cycle contains approximately 10,000 participants and is representative of the United States across all age groups.

### Study Sample

For this analysis, we used NHANES cycles between 2009 and 2014 that comprise information from periodontal examinations. Participants were eligible for periodontal examination if they were aged ≥30 y. Participants who were edentulous and those who had a heart transplant, artificial heart valve, or congenital heart disease were excluded, as were those who required antibiotic prophylaxis prior to examination. To explore multimorbidity, we further excluded participants who did not have a systemic disease diagnosis (Appendix Fig. 1).

### Periodontitis Case Definition

The periodontal examination was conducted at a mobile center by trained dentists who measured clinical attachment loss (CAL) and probing depth across 6 interproximal sites per tooth, excluding third molars. Data were inputted by a health technician. The interclass correlation coefficients of mean attachment loss were ≥0.80 for the included cycles and were in the main more reliable than probing depth measurements. We used the proportion of sites with CAL ≥ 3 mm as a measure of extent of periodontitis, which we split into 4 equal quartiles (Wright et al. 2020). We allocated each participant into 1 of the 4 quartiles according to one’s CAL measures. Quartile 1 represents people with mild/less severe periodontitis, while quartile 4 represents people with more severe periodontitis.

### Systemic Disease

A range of systemic diseases was determined by collating participant responses to the question “Have you even been told by a doctor or health professional that you have . . . ?” followed by 1 of the following diseases: diabetes, cancer, heart attack, coronary heart disease, congestive heart failure, angina and stroke, hypertension, arthritis, liver disease, osteoporosis, thyroid disorder, emphysema, and bronchitis. Obesity was defined by body mass index ≥30 kg/m^2^ (National Institutes of Health 1998). Data on osteoporosis were collected only in NHANES cycles 2009 to 2010 and 2013 to 2014.

### Statistical Analysis

Participant characteristics are presented as mean and standard deviation for continuous variables and frequency and percentage for categorical variables. We reported the overall characteristics for the study population and in people across CAL quartiles. As multimorbidity is highly associated with age and sex, we performed 1:1 matching for age and sex across CAL quartiles to ensure that any systemic multimorbidity clusters were not attributed to the difference in age and sex.

**Figure 1.**
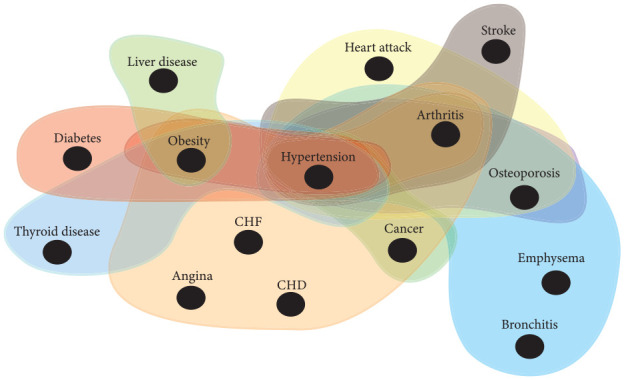
Common systemic multimorbidity clusters identified through hypergraph analysis in people with periodontitis. Nodes represent diseases, and hyperedges (shaded areas) represent multimorbidity clusters. For illustrative purposes only; not all systemic multimorbidity clusters are shown. CHF, congestive heart failure; CHD, coronary heart disease.

Hypergraphs are based on an artificial intelligence output of network analysis to illustrate and explore hyperedges and the hypernodes within them. In a hypergraph, 1 edge can join an infinite number of nodes at once, as opposed to traditional graphs whereby an edge connects only 2 nodes. This technique has demonstrated its utility for illustrating multimorbidity clusters in large-scale data such as electronic health records (Rafferty et al. 2021). Nodes (*N* denotes number of nodes) in a hypergraph represent single diseases, and edges (*E* denotes number of edges) represent a disease set (multimorbidity cluster; Fig. 1). An incidence matrix (*M*) with dimension *N* × *E* represents an unweighted hypergraph. The adjacency matrix (*A*) can be derived from an incidence matrix to allow for further metrics, such as centrality. The adjacency matrix *A* can be calculated as *A* = *M^T^* *M* – *D_N_*, where *T* denotes the transpose and *D_N_* is the degree to which the node is connected to any number of edges, with zeros on the diagonal representing that a node cannot be connected to itself.

To ensure accurate representation of the study sample, the edges of hypergraphs were weighted by *W_E_*, which represents a measure of the number of people who have the disease conditions that are connected to the edge. To account for overlapping sets, in this study the overlap weighting coefficient (*W_E_*) was calculated by the number of people with all the diseases in the set divided by the minimum number of people with 1 of the diseases. Adding weights to the hypergraph results in the following formula for the adjacency matrix: *A* = *M^T^* *W_E_M* – *D_N_*.

The dual hypergraph is symmetrical to a hypergraph; however, the dual hypergraph allows for centrality of multimorbidity clusters to be measured—that is, the degree to which nodes are connected to each multimorbidity cluster. The adjacency matrix (A*) of the dual hypergraph is weighted by the overlap weighting coefficient (*W_E_*) and the number within the cohort with a disease (*W_N_*, or crude prevalence): $A*=\sqrt{W_{E}(}MW_{N}M^{T}-D_{E})\sqrt{W_{E}}.$

The hypergraph can be analyzed via centrality metrics. In accordance with graph theory, the adjacency matrix of the hypergraph represents the weighted strength of connections within the hypergraph (Rafferty et al. 2021). To compute the eigenvector centrality, we calculate the eigenvectors of the adjacency matrix *A*. Eigenvector centrality of the adjacency matrix A refers to the degree in which a given node (disease) is connected to other nodes with their own levels of connectivity. For example, a higher centrality value suggests that a disease cluster is more common than other disease clusters with lower centrality. We applied centrality metrics (eigenvector) and a dual hypergraph (whereby the nodes and edges in the hypergraph are alternated) to the adjacency matrix to determine the most central disease and systemic multimorbidity clusters across CAL quartiles. In addition to eigenvector centrality, we plotted heat maps to illustrate the co-occurrences of 2 single diseases (*W_N_*) and bar plots to show the highest weighted systemic multimorbidity clusters (*W_E_*).

Further to the analysis as applied to the whole study population, we stratified the data by ethnicity, smoking status, and household income to show the differences to single-disease centrality of systemic multimorbidity clusters in these subgroups. We also examined the effect of missing survey responses and osteoporosis information (NHANES cycle 2011 to 2012) by sensitivity analysis in which we compared the results with this cycle removed. Data management, analysis, and hypergraph analysis were conducted in R version (4.0.0; R Core Team 2017). This report conforms to STROBE guidelines.

## Results

### Study Population Characteristics

A total of 6,542 participants were eligible for inclusion across 3 NHANES cycles (2009 to 2014; Appendix Fig. 1). After age and sex matching across the CAL quartiles, 3,736 participants were in the hypergraph analysis. The average age of participants in the matched study cohort was 58.16 y (SD: 13.22), and there were 2,156 (57.7%) females. The proportion with a history of smoking was higher in the highest CAL quartile (55.8%) than in the lowest (40.7%). The most prevalent diseases in the overall study population were arthritis (47.6%), hypertension (63.9%), and obesity (45.9%; Table 1). Population characteristics of the unmatched cohort can be found in Appendix Table 1.

**Table 1.** Characteristics of People by CAL Quartile.

		Proportion CAL ≥ 3 mm, Quartile			
Characteristic^a^	Overall (*N* = 3,736)	1 (*n* = 934)	2 (*n* = 934)	3 (*n* = 934)	4 (*n* = 934)
Sex: female	2,156 (57.7)	539 (57.7)	539 (57.7)	539 (57.7)	539 (57.7)
Age, y	58.16 (13.22)	58.16 (13.23)	58.16 (13.23)	58.16 (13.23)	58.16 (13.23)
BMI	30.57 (7.18)	30.97 (7.22)	30.48 (6.66)	30.61 (7.50)	30.21 (7.31)
Ethnicity					
White	1,723 (46.1)	540 (57.8)	435 (46.6)	399 (42.7)	349 (37.4)
Other race	2,013 (53.9)	394 (42.2)	499 (53.4)	535 (57.3)	585 (62.6)
Household income, quintile					
5	887 (26.3)	331 (38.4)	238 (28.1)	194 (23.0)	124 (15.0)
4	703 (20.8)	184 (21.4)	189 (22.3)	182 (21.6)	148 (17.9)
3	740 (21.9)	167 (19.4)	175 (20.7)	194 (23.0)	204 (24.7)
2	517 (15.3)	101 (11.7)	120 (14.2)	135 (16.0)	161 (19.5)
1	530 (15.7)	78 (9.1)	125 (14.8)	138 (16.4)	189 (22.9)
Systolic blood pressure	128.27 (18.44)	127.06 (17.54)	126.89 (17.93)	128.15 (18.53)	130.98 (19.43)
Diastolic blood pressure	71.03 (13.48)	71.46 (13.10)	70.94 (12.95)	70.74 (13.44)	70.99 (14.40)
Smoker	1,770 (47.4)	380 (40.7)	391 (41.9)	479 (51.3)	520 (55.8)
Condition					
Angina	112 (3.0)	29 (3.1)	30 (3.2)	31 (3.3)	22 (2.4)
Arthritis	1,775 (47.6)	452 (48.5)	465 (49.8)	443 (47.5)	415 (44.6)
Bronchitis	309 (8.3)	57 (6.1)	73 (7.8)	97 (10.4)	82 (8.8)
Cancer	592 (15.9)	171 (18.3)	148 (15.8)	145 (15.5)	128 (13.7)
CHD	168 (4.5)	42 (4.5)	44 (4.7)	35 (3.8)	47 (5.0)
CHF	131 (3.5)	26 (2.8)	26 (2.8)	40 (4.3)	39 (4.2)
Diabetes	784 (21.8)	174 (19.4)	172 (19.2)	205 (23.0)	233 (25.6)
Emphysema	84 (2.3)	12 (1.3)	16 (1.7)	29 (3.1)	27 (2.9)
Heart attack	184 (4.9)	42 (4.5)	42 (4.5)	42 (4.5)	58 (6.2)
Hypertension	2,383 (63.9)	599 (64.2)	583 (62.6)	577 (61.8)	624 (66.9)
Liver	239 (6.4)	58 (6.2)	57 (6.1)	64 (6.9)	60 (6.4)
Obese	1,696 (45.9)	438 (47.5)	435 (46.9)	418 (45.2)	405 (43.9)
Osteoporosis	265 (11.0)	63 (9.9)	77 (12.7)	75 (12.5)	50 (8.7)
Stroke	193 (5.2)	54 (5.8)	38 (4.1)	43 (4.6)	58 (6.2)
Thyroid	704 (18.9)	203 (21.8)	173 (18.6)	179 (19.2)	149 (16.0)

Values are presented as No. (%) for categorial values and mean (SD) for continuous values.

BMI, body mass index; CAL, clinical attachment loss; CHD, coronary heart disease; CHF, congestive heart failure.

a Means and percentages are calculated for variables excluding missing data. There were missing data in the following variables: BMI (1.0%), blood pressure (3.0%), household income (9.6%), smoking (0.1%), diabetes (3.8%), hypertension (0.1%), arthritis (0.2%), CHF (0.2%), CHD (0.4%), angina (0.3%), heart attack (0.1%), stroke (0.1%), emphysema (0.1%), bronchitis (0.2%), liver disease (0.2%), thyroid disease (0.3%), osteoporosis (35.4%), and obesity (1.0%).

A total of 106 unique systemic multimorbidity clusters were identified. Figure 1 illustrates a hypergraph with nodes representing diseases and hyperedges showing some of the most common systemic multimorbidity clusters identified in the population.

### Single Diseases

The most frequent co-occurrent diseases illustrated in the heat maps by darker green/blue were arthritis, hypertension, and obesity (Appendix Fig. 2). The eigenvector centralities of the hypergraph show that hypertension was the most central disease in the overall population (centrality [C]: 0.50), followed closely by arthritis (C: 0.45) and obesity (C: 0.42). Diabetes appeared more central in the highest CAL quartile (C: 0.31) than the lowest (C: 0.26; Table 2).

**Table 2.** Centrality Values for Single Diseases in the Overall Population by CAL Quartile.

		Proportion CAL ≥ 3 mm, Quartile			
	Overall	1	2	3	4
Hypertension	0.50	0.51	0.50	0.48	0.51
Arthritis	0.45	0.45	0.45	0.45	0.45
Obese	0.42	0.43	0.42	0.41	0.42
Diabetes	0.29	0.26	0.25	0.30	0.31
Thyroid disease	0.21	0.23	0.25	0.19	0.19
Cancer	0.20	0.21	0.25	0.19	0.19
Bronchitis	0.18	0.14	0.18	0.22	0.19
CHD	0.17	0.19	0.17	0.17	0.15
Heart attack	0.17	0.18	0.14	0.17	0.19
CHF	0.15	0.15	0.15	0.17	0.14
Angina	0.14	0.16	0.13	0.16	0.12
Stroke	0.14	0.12	0.15	0.14	0.14
Osteoporosis	0.14	0.14	0.14	0.16	0.10
Emphysema	0.12	0.10	0.13	0.12	0.12
Liver disease	0.11	0.10	0.11	0.12	0.10

CAL, clinical attachment loss; CHD, coronary heart disease; CHF, congestive heart failure.

Stratification by individual factors showed in races other than White that the centrality of arthritis was lower in the lowest CAL quartile (White, C: 0.45; other races, C: 0.38) and the highest (White, C: 0.48; other races, C: 0.43). In the highest CAL quartile, diabetes and stroke were more central to systemic multimorbidity clusters in nonsmokers (diabetes, C: 0.40; stroke, C: 0.25) than smokers (diabetes, C: 0.29; stroke, C: 0.13). These differences were not observed in the lower CAL quartile. Differences between low and high household income were negligible (Table 3).

**Table 3.** Centrality Values and Their Rank in the First and Fourth CAL Quartiles Stratified by Individual Factors.

	Quartile 1						Quartile 4					
	Ethnicity		Smoking		Household Income		Ethnicity		Smoking		Household Income	
	White	Other Race	Smokers	NS	Low	High	White	Other Race	Smokers	NS	Low	High
Hypertension	0.49	0.51	0.50	0.50	0.50	0.49	0.51	0.52	0.50	0.54	0.51	0.53
Arthritis	0.45	0.38	0.46	0.44	0.47	0.42	0.48	0.43	0.47	0.35	0.47	0.41
Obese	0.40	0.48	0.42	0.44	0.43	0.41	0.41	0.42	0.42	0.45	0.41	0.42
Cancer	0.28	0.27	0.24	0.25	0.20	0.32	0.23	0.18	0.18	0.16	0.17	0.26
Thyroid disease	0.27	0.18	0.22	0.29	0.24	0.27	0.21	0.20	0.17	0.18	0.20	0.16
Diabetes	0.23	0.26	0.28	0.27	0.24	0.26	0.27	0.34	0.29	0.40	0.30	0.35
Bronchitis	0.19	0.22	0.19	0.12	0.19	0.17	0.17	0.22	0.21	0.12	0.20	0.16
CHD	0.17	0.10	0.15	0.16	0.17	0.15	0.16	0.13	0.18	0.09	0.16	0.12
CHF	0.15	0.11	0.14	0.16	0.16	0.13	0.14	0.12	0.13	0.13	0.14	0.11
Emphysema	0.14	0.13	0.12	—^a^	0.13	0.12	0.12	0.12	0.13	0.14	0.12	0.12
Osteoporosis	0.14	0.11	0.14	0.13	0.14	0.13	0.11	0.09	0.11	0.08	0.10	0.12
Stroke	0.14	0.14	0.16	0.15	0.15	0.15	0.13	0.14	0.13	0.25	0.14	0.14
Angina	0.13	0.09	0.11	0.12	0.12	0.12	0.12	0.10	0.12	0.09	0.11	0.13
Heart attack	0.12	0.13	0.14	0.14	0.13	0.12	0.20	0.18	0.19	0.15	0.19	0.18
Liver disease	0.10	0.21	0.12	0.11	0.09	0.14	0.09	0.14	0.11	0.09	0.11	0.13

Quartiles based on proportion CAL ≥ 3 mm.

CAL, clinical attachment loss; CHD, coronary heart disease; CHF, congestive heart failure; NS, nonsmokers.

a Insufficient data available.

### Multimorbidity Clusters

**Figure 2.**
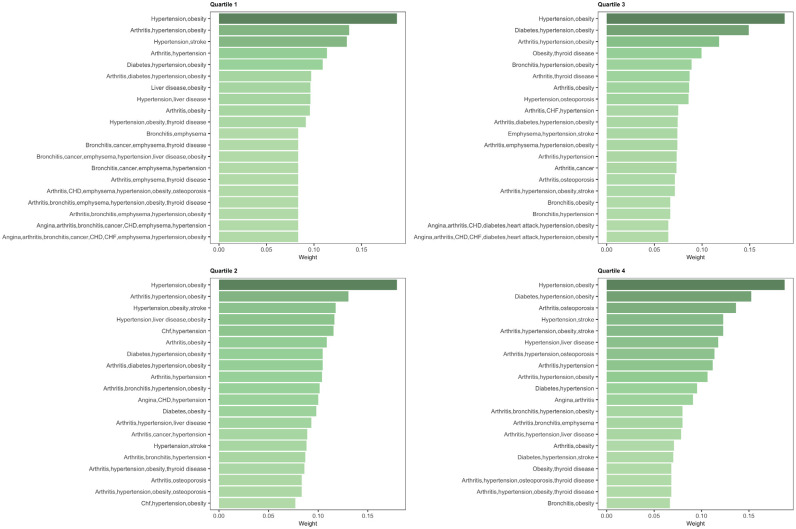
Bar plots for the weights of multimorbidity clusters, stratified by clinical attachment loss quartiles. CHF, congestive heart failure; CHD, coronary heart disease.

“Hypertension, obesity” was the largest weighted systemic multimorbidity cluster across all CAL quartiles. There was a more even distribution in weights of systemic multimorbidity clusters in the highest CAL quartile than the lowest (Fig. 2). The overall centrality values of systemic multimorbidity clusters across CAL quartiles were similar, and the most central clusters contained hypertension and obesity (Appendix Table 2).

### Sensitivity Analysis

Removal of the 2011–2012 cycle, which did not include the question on osteoporosis diagnosis, did not have a notable effect on single-disease or cluster centrality values (Appendix Tables 3 and 4). Further analysis replacing missing diagnoses to positive cases did not show significant difference to the main findings (Appendix Tables 5 and 6).

## Discussion

### Main Findings

In this study, we applied hypergraph analysis to a large cross-sectional cohort to identify the systemic multimorbidity clusters in people with periodontitis. Hypertension, arthritis, and obesity were the single diseases most central/influential to multimorbidity clusters for people with periodontitis; the most frequent systemic multimorbidity cluster was “hypertension, obesity.” Diabetes was more central in participants with a higher degree of CAL, and systemic multimorbidity clusters were more evenly weighted in these participants as compared with those who had lower levels of CAL. Subgroup analysis showed that diabetes and stroke were more central to systemic multimorbidity clusters in nonsmoking participants with higher CAL as compared with smokers, and this difference was not observed in the lower quartile of CAL.

### Synthesis with Previous Studies

The focus of this study was to identify systemic multimorbidity clusters that present in people with periodontitis by using the proportion of CAL ≥ 3 mm and its quartiles as an indicator for the extent of periodontitis. Previous studies mainly focused on the association between periodontitis and only 1 of the systemic conditions, such as cardiovascular disease (Larvin et al. 2021a), hypertension (Czesnikiewicz-Guzik et al. 2019), diabetes (Winning et al. 2017), and COVID-19 outcomes (Larvin et al. 2020; Larvin, Wilmott, et al. 2021). Some studies demonstrated that periodontitis is coprevalent with several systemic conditions, such as obesity, diabetes, and cardiovascular disease, but these were predefined at study inclusion (Kang et al. 2019; Bilgin Çetin et al. 2020; Takeda et al. 2021). Through use of hypergraph analysis as a data-driven technique, our study has identified some common systemic multimorbidity clusters in people with periodontitis and the most influential single diseases to multimorbid presentation. For example, we identified “hypertension, obesity” as the most frequent multimorbidity cluster in people with periodontitis. Hypertension and obesity are 2 common conditions known to usually precede development of further systemic disease due to the inflammatory stresses that they trigger on the body (Liu and Nikolajczyk 2019; Ruan and Gao 2019). Past findings based on cluster analysis also showed that the prevalence of hypertension is common in systemic multimorbidity clusters of the general population (Formiga et al. 2013; Zemedikun et al. 2018). Cluster analyses are historically limited, as they group conditions by how closely related they are, rather than identify tangible systemic multimorbidity clusters as in hypergraph analyses. Our study highlighted that hypertension and obesity are central to multimorbidity in people with periodontitis, and this could be important to develop more targeted treatment intervention for this particular population.

Furthermore, our study has demonstrated that diabetes is more central in multimorbidity clusters for people with severe periodontitis versus people with mild periodontitis. This finding aligns with suggestions of a bidirectional relationship between periodontitis and diabetes (Casanova et al. 2014; Stöhr et al. 2021). The systemic inflammation associated with severe periodontitis likely disrupts immune function and glucose handling associated with diabetes and vice versa (Donath et al. 2019; Berbudi et al. 2020). The European Federation of Periodontology and the International Diabetes Federation have developed a roadmap for health care professionals in managing these patients with collaborative care pathways (Sanz et al. 2018). Our findings advocate consideration of the wider systemic multimorbidity clusters in which these patients may also present; specifically, such clusters should be reflected in the recent classification of periodontal disease as separate diagnostic entities.

No previous studies have systematically investigated the systemic multimorbidity clusters in people with periodontitis, particularly the impact of individual factors on clustering patterns. To account for the high correlation between multimorbidity and age and sex, we performed 1:1 matching across CAL quartiles to ensure that identified multimorbidity clusters were independent of age and sex. In addition, we assessed the impact of ethnicity, smoking status, and deprivation on multimorbidity clusters. We found that ethnicity and deprivation affect the clustering pattern of arthritis and obesity more in people with mild periodontitis, while smoking status and deprivation affect the clustering pattern of diabetes and cancer in people with severe periodontitis. These findings highlight the difference in multimorbidity clusters by patient factors, and such difference should be taken into account when designing and planning health care provision and strategies for disease prevention.

### Strengths and Limitations

As the first to use hypergraph analysis in oral health research, our study has notable strengths. The hypergraph technique is in its relative infancy within the health research sector and is showing great promise in using cross-sectional data as a nonintrusive information source and to move beyond descriptive analysis, revealing more about patient multimorbid presentation. Hypergraph analysis for multimorbidity research is distinct from traditional regression methods as it uses the data to identify multimorbidity clusters of several diseases simultaneously. Previous regression analyses are limited to single predefined outcomes and pairwise methods to surmise multimorbidity. Our study has demonstrated the utility of this technique in oral health research, as a means of using big data to identify and compare tangible systemic multimorbidity clusters in populations with periodontitis. As awareness of multimorbidity in dental patients is coming to the forefront of oral health research (Watt and Serban 2020), our findings improve understanding of multimorbidity in people periodontitis that may present to the dental clinic. Another strength of our study is the clinical periodontal examination data including CAL, which enables exploration of periodontitis severity with clinical validation and high intraclass correlation (Dye et al. 2019). Furthermore, as a representative national survey across multiple years, the results from NHANES data can provide improved generalizability than typical epidemiologic studies that can be limited by selection bias.

Our study was limited to 3 NHANES cycles due to the availability of periodontal examination data, which limited our sample size; future NHANES cycles with periodontal examination could supplement our findings with more data and more robust findings. The NHANES data set does not supply linked electronic health records; therefore, our analysis relied on self-reported multimorbidity. As NHANES was a cross-sectional national survey, we could not ascertain whether periodontitis occurred before or after the multimorbidity clusters, because the timing of disease diagnosis was not available. We used quartiles of CAL ≥ 3 mm as a surrogate for a periodontitis case definition. While this ensured equal sample sizes across groups, it should be noted that clinical guidelines recommend CAL and probing depth for periodontitis classification (Caton et al. 2018).

## Conclusion

This study has revealed a range of common systemic multimorbidity clusters in people with periodontitis. People with periodontitis are more likely to present with hypertension and obesity together, and these conditions are highly influential to the presence of other multimorbidity. In addition, diabetes is more influential to multimorbidity clusters in people with severe periodontitis. Patient factors such as deprivation and smoking status could influence the pattern of multimorbidity clusters.

## Author Contributions

H. Larvin, contributed to conception, data analysis, and interpretation, drafted and critically revised the manuscript; J. Kang, V.R. Aggarwal, S. Pavitt, contributed to data interpretation, critically revised the manuscript; J. Wu, contributed to conception, design, data acquisition, and interpretation, critically revised the manuscript. All authors gave final approval and agree to be accountable for all aspects of the work.

## Supplemental Material

sj-docx-1-jdr-10.1177_00220345221098910 – Supplemental material for Systemic Multimorbidity Clusters in People with Periodontitis Click here for additional data file. Supplemental material, sj-docx-1-jdr-10.1177_00220345221098910 for Systemic Multimorbidity Clusters in People with Periodontitis by H. Larvin, J. Kang, V.R. Aggarwal, S. Pavitt and J. Wu in Journal of Dental Research
