# Supplementary material for: Systemic Multimorbidity Clusters in People with Periodontitis
Source: J Dent Res. 2022 Jun 9;101(11):1335–42. doi: 10.1177/00220345221098910 (PMC9516606; doi:10.1177/00220345221098910)
Supplement: sj-docx-1-jdr-10.1177_00220345221098910 – Supplemental material for Systemic Multimorbidity Clusters in People with Periodontitis [file sj-docx-1-jdr-10.1177_00220345221098910.docx]

**Supplement**

**Table S1.** The characteristics of people by CAL quartile, unmatched cohort.

|  | |  | |  | **Proportion CAL** $\boldsymbol{\geq}$ **3 mm, quartile** | | | |
| --- | --- | --- | --- | --- | --- | --- | --- | --- |
|  | |  | | **Overall**  (n = 6542) | 1  (n = 1636) | 2  (n = 1636) | 3  (n = 1635) | 4  (n = 1635) |
| Sex, female (%) | | | | 3513 (53.7) | 1101 (67.3) | 975 (59.6) | 843 (51.6) | 594 (36.3) |
| Age, mean (SD) | | | | 57.52 (13.89) | 52.10 (14.48) | 57.43 (13.43) | 60.36 (13.18) | 60.20 (12.80) |
| BMI, mean (SD) | | | | 30.40 (7.18) | 31.12 (7.77) | 30.49 (6.86) | 30.23 (7.19) | 29.77 (6.80) |
| Ethnicity (%) | | White | | 3017 (46.1) | 908 (55.5) | 758 (46.3) | 721 (44.1) | 630 (38.5) |
|  | | Other race | | 3525 (53.9) | 728 (44.5) | 878 (53.7) | 914 (55.9) | 1005 (61.5) |
| Household income, quintile (%) | | | 5 | 1568 (26.4) | 558 (36.7) | 424 (28.6) | 340 (22.9) | 246 (16.9) |
|  |  |  | 4 | 1223 (20.6) | 310 (20.4) | 310 (20.9) | 313 (21.1) | 290 (19.9) |
|  |  |  | 3 | 1320 (22.2) | 295 (19.4) | 320 (21.6) | 355 (23.9) | 350 (24.0) |
|  |  |  | 2 | 948 (16.0) | 202 (13.3) | 222 (15.0) | 239 (16.1) | 285 (19.6) |
|  |  |  | 1 | 884 (14.9) | 155 (10.2) | 204 (13.8) | 239 (16.1) | 286 (19.6) |
| Systolic blood pressure, mean (SD) | | | | 128.02 (18.77) | 124.24 (17.59) | 126.88 (17.91) | 129.53 (19.31) | 131.47 (19.42) |
| Diastolic blood pressure, mean (SD) | | | | 71.03 (13.50) | 71.82 (12.48) | 71.33 (13.13) | 70.29 (13.58) | 70.67 (14.70) |
| Smoker (%) | | | | 3124 (47.8) | 630 (38.5) | 695 (42.5) | 833 (50.9) | 966 (59.2) |
| **Conditions** | | | |  |  |  |  |  |
|  | Angina (%) | | | 206 (3.2) | 39 (2.4) | 42 (2.6) | 70 (4.3) | 55 (3.4) |
|  | Arthritis (%) | | | 2977 (45.6) | 709 (43.4) | 777 (47.6) | 790 (48.4) | 701 (43.1) |
|  | Bronchitis (%) | | | 549 (8.4) | 137 (8.4) | 144 (8.8) | 147 (9.0) | 121 (7.4) |
|  | Cancer (%) | | | 1045 (16.0) | 254 (15.5) | 262 (16.0) | 276 (16.9) | 253 (15.5) |
|  | CHD (%) | | | 327 (5.0) | 50 (3.1) | 72 (4.4) | 91 (5.6) | 114 (7.0) |
|  | CHF (%) | | | 242 (3.7) | 34 (2.1) | 52 (3.2) | 78 (4.8) | 78 (4.8) |
|  | Diabetes (%) | | | 1387 (22.0) | 258 (16.4) | 320 (20.3) | 377 (24.0) | 432 (27.4) |
|  | Emphysema (%) | | | 155 (2.4) | 21 (1.3) | 27 (1.7) | 45 (2.8) | 62 (3.8) |
|  | Heart attack (%) | | | 343 (5.2) | 49 (3.0) | 71 (4.3) | 100 (6.1) | 123 (7.5) |
|  | Hypertension (%) | | | 4134 (63.2) | 947 (57.9) | 1012 (62.0) | 1056 (64.6) | 1119 (68.5) |
|  | Liver (%) | | | 417 (6.4) | 100 (6.1) | 91 (5.6) | 114 (7.0) | 112 (6.9) |
|  | Obese (%) | | | 2934 (45.2) | 789 (48.7) | 768 (47.3) | 717 (44.3) | 660 (40.7) |
|  | Osteoporosis (%) | | | 426 (10.1) | 98 (9.2) | 129 (12.1) | 130 (12.3) | 69 (6.7) |
|  | Stroke (%) | | | 325 (5.0) | 75 (4.6) | 60 (3.7) | 90 (5.5) | 100 (6.1) |
|  | Thyroid (%) | | | 1162 (17.8) | 373 (22.9) | 298 (18.2) | 285 (17.5) | 206 (12.6) |

K**ey:** body mass index (BMI), clinical attachment loss (CAL), coronary heart disease (CHD), congestive heart failure (CHF), number of participants (n), standard deviation (SD)
**Note:** means and percentages are calculated for variables excluding missing data. There was missing data in the following variables: BMI (0.9%), blood pressure (2.8%), household income (9.2%), diabetes (3.8%), hypertension (0.1%), arthritis (0.2%), CHF (0.3%), CHD (0.5%), angina (0.3%), heart attack (0.1%), stroke (0.1%), emphysema (0.1%), bronchitis (0.2%), liver disease (0.2%), thyroid disease (0.2%), osteoporosis (35.4%), cancer (0.1%), obesity (0.9%).

**Table S2.** Largest centrality values for multimorbidity clusters in the overall population, and by CAL quartile.

|  |  | **Proportion CAL** $\boldsymbol{\geq}$ **3 mm, quartile** | | | |
| --- | --- | --- | --- | --- | --- |
|  | **Overall** | 1 | 2 | 3 | 4 |
| Arthritis, hypertension, obesity, stroke | 0.12 | 0.07 | * | 0.13 | 0.16 |
| Arthritis, hypertension, obesity | 0.16 | 0.16 | 0.17 | 0.16 | 0.15 |
| Hypertension, obesity | 0.15 | 0.15 | 0.15 | 0.15 | 0.15 |
| Diabetes, hypertension, obesity | 0.13 | 0.12 | 0.11 | 0.14 | 0.14 |
| Arthritis, bronchitis, hypertension, obesity | 0.13 | 0.11 | 0.15 | 0.10 | 0.13 |
| Arthritis, diabetes, hypertension, obesity | 0.14 | 0.15 | 0.16 | 0.14 | 0.12 |
| Arthritis, hypertension, obesity, thyroid disease | 0.12 | 0.11 | 0.14 | 0.11 | 0.12 |
| Arthritis, hypertension | 0.11 | 0.11 | 0.11 | 0.10 | 0.12 |
| Arthritis, cancer, diabetes, hypertension, obesity | 0.09 | 0.10 | 0.04 | 0.08 | 0.12 |
| Arthritis, hypertension, osteoporosis | 0.09 | 0.05 | 0.09 | 0.09 | 0.12 |

**Key:** coronary heart disease (CHD), congestive heart failure (CHF), periodontitis (PD), insufficient data available (*).

**Table S3.** Centrality values for single diseases in the overall population, by CAL quartile, with 2011 – 2012 cohort removed.

|  |  | **Proportion CAL** $\boldsymbol{\geq}$ **3 mm, quartile** | | | |
| --- | --- | --- | --- | --- | --- |
|  | **Overall** | 1 | 2 | 3 | 4 |
| Hypertension | 0.50 | 0.50 | 0.51 | 0.46 | 0.52 |
| Arthritis | 0.45 | 0.44 | 0.45 | 0.43 | 0.46 |
| Obese | 0.42 | 0.44 | 0.41 | 0.41 | 0.42 |
| Diabetes | 0.29 | 0.23 | 0.25 | 0.30 | 0.31 |
| Thyroid disease | 0.21 | 0.28 | 0.25 | 0.18 | 0.16 |
| Cancer | 0.20 | 0.24 | 0.19 | 0.20 | 0.18 |
| Bronchitis | 0.19 | 0.16 | 0.16 | 0.22 | 0.18 |
| Heart attack | 0.18 | 0.12 | 0.20 | 0.18 | 0.21 |
| Osteoporosis | 0.18 | 0.18 | 0.19 | 0.20 | 0.11 |
| CHD | 0.16 | 0.14 | 0.19 | 0.17 | 0.17 |
| CHF | 0.15 | 0.13 | 0.13 | 0.18 | 0.15 |
| Angina | 0.14 | 0.10 | 0.16 | 0.17 | 0.10 |
| Stroke | 0.14 | 0.17 | 0.13 | 0.16 | 0.12 |
| Emphysema | 0.12 | 0.15 | 0.10 | 0.13 | 0.11 |
| Liver disease | 0.11 | 0.13 | 0.10 | 0.12 | 0.09 |

**Key:** clinical attachment loss (CAL), coronary heart disease (CHD), congestive heart failure (CHF), periodontitis (PD).

**Table S4.** Largest centrality values for multimorbidity clusters in the overall population, by CAL quartile, with 2011 – 2012 cohort removed.

|  |  | **Proportion CAL** $\boldsymbol{\geq}$ **3 mm, quartile** | | | |
| --- | --- | --- | --- | --- | --- |
|  | **Overall** | 1 | 2 | 3 | 4 |
| Arthritis, hypertension, obesity | 0.16 | 0.15 | 0.16 | 0.17 | 0.16 |
| Hypertension, obesity | 0.15 | 0.15 | 0.15 | 0.15 | 0.15 |
| Arthritis, diabetes, hypertension, obesity | 0.14 | 0.12 | 0.14 | 0.16 | 0.15 |
| Arthritis, bronchitis, hypertension, obesity | 0.13 | 0.13 | 0.10 | 0.15 | 0.11 |
| Diabetes, hypertension, obesity | 0.13 | 0.14 | 0.14 | 0.11 | 0.12 |
| Arthritis, hypertension, obesity, stroke | 0.12 | 0.16 | 0.13 | * | 0.07 |
| Arthritis, hypertension, obesity, thyroid disease | 0.12 | 0.12 | 0.11 | 0.14 | 0.11 |
| Arthritis, hypertension | 0.11 | 0.12 | 0.10 | 0.11 | 0.11 |
| Arthritis, cancer, hypertension, obesity | 0.10 | 0.07 | 0.07 | 0.12 | 0.10 |
| Arthritis, heart attack, hypertension, obesity | 0.10 | 0.11 | * | 0.11 | 0.10 |

**Key:** clinical attachment loss (CAL), coronary heart disease (CHD), congestive heart failure (CHF), periodontitis (PD), insufficient data available (*).

**Table S5.** Centrality values for single diseases in the overall population, by CAL quartile, changing missing diagnoses to positive cases.

|  |  | **Proportion CAL** $\boldsymbol{\geq}$ **3 mm, quartile** | | | |
| --- | --- | --- | --- | --- | --- |
|  | **Overall** | 1 | 2 | 3 | 4 |
| Hypertension | 0.47 | 0.47 | 0.48 | 0.46 | 0.48 |
| Arthritis | 0.42 | 0.41 | 0.42 | 0.42 | 0.42 |
| Obese | 0.40 | 0.39 | 0.40 | 0.40 | 0.39 |
| Osteoporosis | 0.36 | 0.36 | 0.37 | 0.35 | 0.37 |
| Diabetes | 0.29 | 0.27 | 0.27 | 0.30 | 0.29 |
| Thyroid disease | 0.20 | 0.21 | 0.20 | 0.18 | 0.19 |
| Cancer | 0.18 | 0.22 | 0.19 | 0.17 | 0.17 |
| Bronchitis | 0.17 | 0.16 | 0.14 | 0.21 | 0.18 |
| CHD | 0.15 | 0.17 | 0.17 | 0.15 | 0.14 |
| Heart attack | 0.15 | 0.14 | 0.16 | 0.15 | 0.16 |
| CHF | 0.14 | 0.14 | 0.15 | 0.16 | 0.14 |
| Angina | 0.13 | 0.12 | 0.15 | 0.15 | 0.11 |
| Stroke | 0.13 | 0.15 | 0.11 | 0.13 | 0.14 |
| Emphysema | 0.11 | 0.12 | 0.10 | 0.12 | 0.11 |
| Liver disease | 0.11 | 0.10 | 0.10 | 0.11 | 0.10 |

**Key:** clinical attachment loss (CAL), coronary heart disease (CHD), congestive heart failure (CHF), periodontitis (PD).

**Table S6.** Largest centrality values for multimorbid clusters in the overall population, by CAL quartile, changing missing diagnoses to positive cases.

|  |  | **Proportion CAL** $\boldsymbol{\geq}$ **3 mm, quartile** | | | |
| --- | --- | --- | --- | --- | --- |
|  | **Overall** | 1 | 2 | 3 | 4 |
| Arthritis, hypertension, obesity | 0.16 | 0.15 | 0.16 | 0.17 | 0.16 |
| Hypertension, obesity | 0.15 | 0.15 | 0.15 | 0.15 | 0.15 |
| Arthritis, diabetes, hypertension, obesity | 0.14 | 0.12 | 0.14 | 0.16 | 0.15 |
| Arthritis, bronchitis, hypertension, obesity | 0.13 | 0.13 | 0.10 | 0.15 | 0.11 |
| Diabetes, hypertension, obesity | 0.13 | 0.14 | 0.14 | 0.11 | 0.12 |
| Arthritis, hypertension, obesity, stroke | 0.12 | 0.16 | 0.13 | * | 0.07 |
| Arthritis, hypertension, obesity, thyroid disease | 0.12 | 0.12 | 0.11 | 0.14 | 0.11 |
| Arthritis, hypertension | 0.11 | 0.12 | 0.10 | 0.11 | 0.11 |
| Arthritis, cancer, hypertension, obesity | 0.10 | 0.07 | 0.07 | 0.12 | 0.10 |
| Arthritis, heart attack, hypertension, obesity | 0.10 | 0.11 | * | 0.11 | 0.10 |

**Key:** clinical attachment loss (CAL), coronary heart disease (CHD), congestive heart failure (CHF), periodontitis (PD), insufficient data available (*).

**
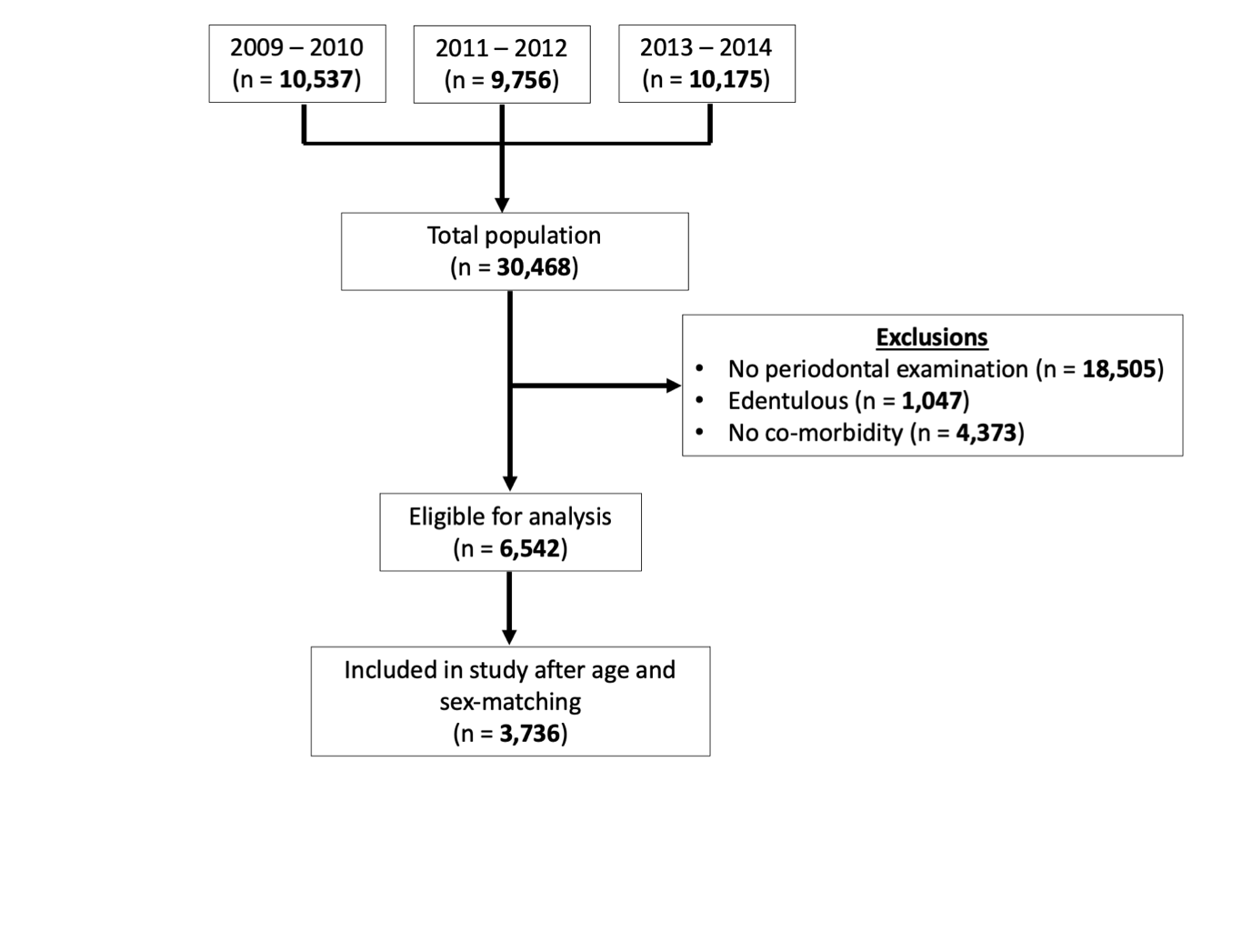
**

**Figure S1** Flow chart of study population.

**Key:** number of participants (n).

**Figure S2** Heatmaps of weights between diseases across all multimorbidity clusters, stratified by quartiles for proportion of clinical attachment loss > 3mm.

**Key:** coronary heart disease (CHD), congestive heart failure (CHF).
